# Six Years Follow-up of the Levels of TNF-*α* and IL-6 in Patients with Complex Regional Pain Syndrome Type 1

**DOI:** 10.1155/2008/469439

**Published:** 2008-06-25

**Authors:** Feikje Wesseldijk, Frank J. P. M. Huygen, Claudia Heijmans-Antonissen, Sjoerd P. Niehof, Freek J. Zijlstra

**Affiliations:** ^1^Department of Anesthesiology, Pain Treatment Center, Erasmus MC, P.O. Box 2040, 3000 CA Rotterdam, The Netherlands; ^2^Department of Anesthesiology, Experimental Anesthesiology, Erasmus MC, P.O. Box 2040, 3000 CA Rotterdam, The Netherlands

## Abstract

In an earlier study, levels of the proinflammatory cytokines TNF-*α* and IL-6 are higher in blisters fluid from the complex regional pain syndrome type 1 (CRPS1) side obtained at 6 and 30 months (median) after the initial event. The aim of this follow-up study is to determine the involvement of these cytokines in long lasting CRPS1. Twelve CRPS1 patients, with median disease duration of 72 months, participated. The levels of TNF-*α* and IL-6 were measured in blister fluid; disease activity was reevaluated by measuring pain and differences in temperature, volume, and mobility between both extremities. Differences in levels of IL-6 and TNF-*α* and mobility between both sides were significantly decreased. Pain and differences in temperature and volume were not significantly altered. No correlation was found between the cytokines and the disease characteristics. These results indicate that IL-6 and TNF-*α* are only partially responsible for the signs and symptoms of CRPS1.

## 1. INTRODUCTION

Complex regional pain syndrome type 1 (CRPS1) is a disease of an extremity that usually occurs as a complication after surgery or trauma, although spontaneous occurrence is also described [1]. 

The pathophysiology of CRPS1 is still not totally clear. In general, three mechanisms are thought to be involved: afferent mechanisms (e.g., neurogenic inflammation) [2–4], efferent mechanisms (e.g., autonomic disturbances) [5], and central nervous system mechanisms (e.g., cerebral plasticity) [6]. Based on our review of the literature regarding the CRPS pathophysiology, we hypothesized that following a trauma or surgery, the normal sterile inflammatory response runs out of control, and is perhaps initiated by a genetic and/or acquired immunologic disorder [7]. Neuroimmune activation of cells in the peripheral nervous system, which is part of the afferent mechanism, apparently results in central sensitization and exacerbation of pain [8]. Neuropeptides, cytokines, and other mediators are released during the inflammation [9, 10] and cause the prominent signs and symptoms, which resemble inflammation; these include increased skin temperature, edema, pain, loss of function, and redness [7, 11].

Levels of TNF-*α* and IL-6 were previously shown to be elevated in fluid of artificially induced skin blisters from the CRPS1 side in the initial stage of the disease [9, 12, 13]. These observations suggest local inflammation. We hypothesized that in most patients local inflammation would only be present during the first year of the disease, since the clinical signs and symptoms of CRPS1 are expected to diminish over time in most patients. Therefore, we predicted that the formation of proinflammatory mediators (such as IL-6 and TNF-*α*) should decline during the course of the disease. In an earlier study, we showed that although the levels of TNF-*α* and IL-6 declined in the intermediate stage of the disease, they were still significantly elevated in the CRPS1 extremity [13]. In the present study, we examine whether this decline in cytokine levels continues during the course of the disease and whether this decline is correlated with a possible improvement in disease activity as measured by registration of pain, and by the measurement of differences in temperature, volume, and mobility between the CRPS1 and contralateral extremities.

## 2. MATERIALS AND METHODS

The protocol was approved by the medical ethics committee of the Erasmus MC Rotterdam (MEC no. 1989.780/2001/24). Guidelines according to the Declaration of Helsinki (amended version of 2002) and Good Clinical Practice (ICH/GCP version 1996) were followed. Data collection and calculations were performed according to guidelines for registration of personal data.

### 2.1. Patients

Sixty-six patients with CRPS1 in one extremity for 7 months after the initial event participated in several studies conducted between April 2001 and February 2004 (T0) to investigate the pathophysiology of CRPS1 or the effects of specific treatments for CRPS1 [9, 12, 14]. At the time of the first follow-up study in 2004, 25 patients with CRPS1 with a median disease duration of 30 months after the initial event agreed to participate (T1) [13]. In 2007, these 25 patients were again asked to participate. One patient had died, one patient appeared to have CRPS type 2, and 11 patients chose not to participate again. In the end, 12 patients with CRPS1 were included in this study (T2).

For this study, we used the results obtained at baseline (T0), first follow-up measurement (T1), and second follow-up measurement (T2) from only these 12 patients. All 12 patients fulfilled the CRPS1 criteria by Bruehl et al. [15] at the first measurement (T0), performed shortly after the initial event (median 4 months) which resulted in the development of the disease.

### 2.2. Pain assessment

The intensity of pain was assessed by using a visual analoge scale (VAS) recorded in 0–100 millimeters [16]. The McGill Pain Questionnaire, Dutch Language version (MPQ-DLV), was measured by counting the total number of words chosen from 20 items [17].

### 2.3. Temperature measurement

Skin temperature was measured using an infrared tympanic probe thermometer, First Temp Genius (Sherwood Medical, Crawley, Sussex, UK) [18]. Measurements were obtained on the dorsal aspect of the hand in a matrix of five points. The difference in mean temperature between the CRPS1 and contralateral extremities was calculated, and the data were expressed with respect to the temperature of the unaffected hand.

### 2.4. Assessment of volume

Volume was measured with a volumeter, which measures the amount of water displaced by immersion of a body part [19]. The difference between the CRPS1 and contralateral extremities was calculated as a percentage of the contralateral extremity.

### 2.5. Assessment of mobility

Mobility was assessed by measuring the active range of motion (AROM), which is defined as the arc of motion requiring muscle power to achieve the motion of a joint [20, 21]. The AROM on the CRPS1 extremity was multiplied by 100 and divided by the AROM on the contralateral extremity to derive the percentage of normal mobility.

### 2.6. Blisters

Artificial skin blisters were induced using a suction method [9, 14, 22]. A skin suction chamber was positioned on the skin of the CRPS1 and contralateral extremities. A vacuum of 300 mm Hg was applied with an Atmoforte 350 A aspirator pump (ATMOS Medizintechnik, Lenzkirch, Germany). After 15 minutes, the vacuum was reduced to 250 mm Hg, and after another 15 minutes it was reduced to 200 mm Hg. This negative pressure was maintained for 2–2.5 hours. The blisters created were punctured, and fluid was pooled from each side into a 1.5 mL Eppendorf conical polypropylene tube and centrifuged for 5 minutes at 1600 × g. All samples were stored in 1 mL conical polypropylene tubes at −80°C until analysis [9, 22].

### 2.7. Cytokine assays

Blister fluid samples were diluted 4-fold in appropriate calibrator dilutent assay buffer for the direct measurement of cytokines. Cytokine assays were performed following the manufacturer's protocol, PeliKine human ELISA kits for IL-6 [M1906] and TNF-*α* [M1920] (CLB, Amsterdam, the Netherlands). The standard curve ranges and mean calculated zero signal ±3 (SD) were 0–80 and 0.3 pg/mL for IL-6 and 0–1000 and 1 pg/mL for TNF-*α*. The absorbance per well was measured at 450 nm with a Medgenix EASIA reader. Sample concentrations were calculated using the appropriate standard calibration lines and the Softmax software of the reader.

### 2.8. Statistical analysis

Data were analyzed with SPSS for Windows, version 14.0. To determine whether the 12 CRPS1 patients from this study were a representative group of the initial 66 CRPS1 patients who started the follow-up study, the one-way ANOVA test was used for a comparison between groups for the proinflammatory cytokines, and the outcome parameters pain, temperature, volume, and mobility. To determine if the differences in means for these selected parameters were significantly different from 0, we applied the one-sample *t*-test for comparisons of the levels of the proinflammatory cytokines between both extremities, and to the differences in the outcome parameters of temperature, volume, and mobility. Mixed model analysis was used to compare the differences between both extremities to each other for the levels of the cytokines, VAS, McGill, temperature, volume, and mobility between each time point. Correlations between the differences in levels of IL-6 and TNF-*α* and the outcome parameters were calculated using the Pearson correlation test. Significance was accepted at the *P* < .05 (two-sided) level. 

## 3. RESULTS

The group of 12 CRPS1 patients was a good representation of the original group of 66 CRPS1 patients with which we initiated the follow-up study 6 years ago. The groups were equal for difference in levels of IL-6 (*P* = .60) and TNF-*α* (*P* = .72). They were also equal for the McGill pain score (*P* = .41), temperature difference (*P* = .41), volume difference (*P* = .48), and mobility (*P* = .38). The two groups were only significantly different from each other for VAS pain (*P* = .03). The characteristics and medication use of the 12 patients with CRPS1 in one extremity for 6 years (median) who were examined 3 times during the follow-up of their CRPS1 are presented in Table 1.

**Figure 1:**
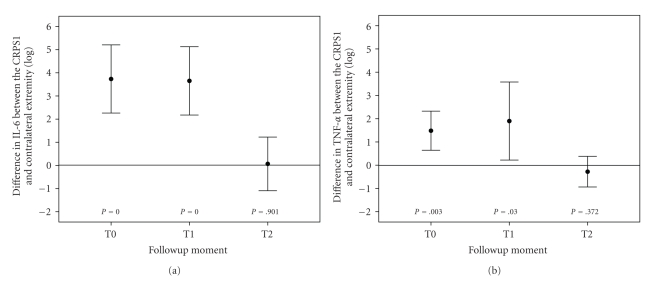
Differences in levels of IL-6 and in TNF-*α* in blister fluid between the CRPS1 and contralateral extremities over the course of CRPS1. Blister fluid was collected as described in materials and methods (see Section 2) and IL-6 (panel a) and TNF-*α* (panel b) levels (pg/mL) in the CRPS1 and the contralateral limbs were measured by ELISA. The data obtained from the same 12 patients at each time period are expressed as the difference in IL-6 or TNF-*α* levels between the two sides (log pg/mL). Each time point shows the mean ± the standard deviation. The *P* values represent the deviation from no difference (0).

The IL-6 and TNF-*α* levels in blister fluid at all time intervals are presented in Table 2. The differences in levels of IL-6 between both extremities did not significantly change at T1 compared to T0 (*P* = .531) and at T2 compared to T0 (*P* = .063). However, the differences in levels did significantly decrease at T2 compared to T1 (*P* = .028). The differences in levels of TNF-*α* between both extremities did not significantly change at T1 compared to T0 (*P* = .892) and at T2 compared to T0 (*P* = .153). The decrease in differences from T1 to T2 almost reached significance (*P* = .064). At the T0 and T1 measurement, the difference in levels of IL-6 and TNF-*α* between the CRPS1 side and the contralateral side in blister fluid was significantly different from 0. However, at the T2 measurement, no significant differences between the two sides were evident (Figures 1(a) and 1(b)) for the 12 CRPS1 patients.

**Figure 2:**
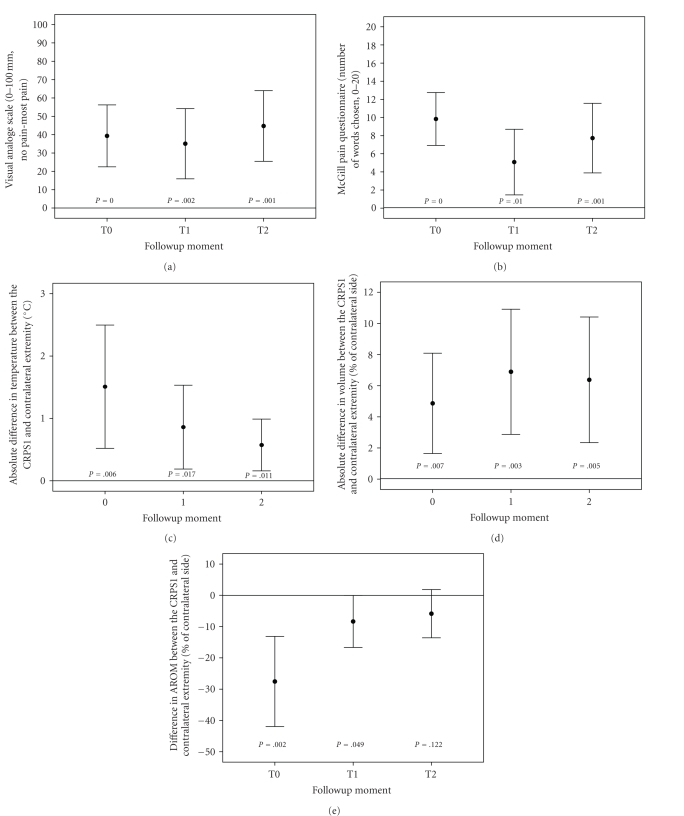
The assessment of pain over the course of CRPS1 disease as measured by the Visual Analogue Scale is shown in panel (a). Panel (b) shows the assessment of pain as measured by the McGill Pain Questionnaire. Panel (c), (d), and (e), respectively, show the absolute differences in skin surface temperature, the absolute differences in volume, and the differences in AROM between the CRPS1 and contralateral sides in CRPS1 patients over the course of disease. The data obtained from the same 12 patients at the 3 points of measurement were collected as described in materials and methods and are expressed as the mean ± standard deviation. The *P* values represent the deviation from no difference (0)

Also the signs and symptoms of impairment were measured at T0, T1, and T2 in terms of pain and differences in temperature, volume, and mobility between the CRPS1 and the contralateral extremities. The VAS pain did not significantly change during the course of the disease in these 12 patients (*P* = .472). The McGill pain score improved significantly at T1 compared to T0 (*P* = .006), but pain was significantly worse at T2 compared to T1 (*P* = .041). Furthermore, no significant improvement was seen at T2 as compared to T0 (*P* = .567). The VAS pain and the McGill pain score significantly differed from 0 during all 6 years of follow-up (Figures 2(a) and 2(b)).

The absolute difference in temperature and volume between both sides did not change during the course of the disease (*P* = .204 and *P* = .509, resp.). The difference between the CRPS1 and contralateral side did vary significantly from 0 at all three moments of measurement for temperature and volume (Figures 2(c) and 2(d)). Mobility improved significantly at T1 and T2 as compared to T0 (*P* = .002 for both), with no significant increase in AROM at T2 compared to T1 (*P* = .353). The difference in mobility between both sides was significantly different from 0 at T0 and T1, but no significant difference was found at T2 (Figure 2(e)).

No correlation was found between the differences in levels of the proinflammatory cytokines and the other disease-related parameters pain and differences between both extremities for temperature, volume, and mobility during the course of the disease.

The total use of disease-related medication was divided in 8 categories (see Table 1). The total number of patients using medication as well as the variety in types of medication decreased during the course of the disease.

## 4. DISCUSSION

In a six years follow-up study in 12 CRPS1 patients, we found a decrease in the extent of the differences in levels of TNF-*α* and IL-6 in blister fluid. After disease duration of 4 months (T0) and of 3 years (T1), the levels of these cytokines were significantly higher in the CRPS1 extremity compared to the contralateral extremity. However, after 6 years (T2) the differences in the cytokine levels between the two extremities were not significantly different. In contrast, the difference in mobility between both sides was significantly improved after 6 years. Pain and the differences in temperature and volume were not significantly altered during the course of the disease. No correlation was found between the proinflammatory cytokines and the disease characteristics.

Because TNF-*α* and IL-6 are proinflammatory cytokines, one might expect that these markers of inflammation would be directly related to the characteristics of inflammation: pain, temperature increase, edema, and loss of function. In this study, the levels of TNF-*α* and IL-6 were diminished during the course of the disease, but no clear improvement of inflammatory signs was found. Most of the 12 patients still reported much pain. The VAS score did not change during the course of the disease, and while the McGill Pain score improved at first, it worsened again at the third measurement. The absolute temperature difference tended to diminish during the course of the disease; however, this was not significant. The volume difference also did not change significantly during the course of the disease. Only the AROM improved significantly after 6 years; the patients only described some stiffness of the joints.

No improvement of the signs and symptoms of inflammation was found as described above, however after six years, a decline in the number of patients using medication that could counteract inflammation has been observed, such as NSAIDs, opiates, and antioxidants. We concluded that the normalization of inflammatory mediators after six years was not affected through pharmacological intervention, but due to a diminution of disease activity.

An explanation for a decrease in differences in the levels of TNF-*α* and IL-6 between both sides during the course of CRPS1 is a spreading of the disease from the CRPS1 side to the contralateral extremity. This spreading could also be explained by the fact that the levels in the CRPS1 side are increased during the course of the disease. However, the increased levels of the contralateral side measured 6 years after the initial event do not even come close to the high levels of TNF-*α* and IL-6 measured in the CRPS1 side 4 months and 3 years after the initial event. Furthermore, the levels of IL-6 of both sides at T2 are within the normal range of the levels measured in blister fluid from healthy controls (mean 16 pg/mL) [23]. These findings do not support the theory of spreading. Finally, one would expect that the differences between both sides in disease activity would decrease during the course of the disease when there is spreading. This is not confirmed in this follow-up study.

In the present study, in contrast with the diminished levels of the proinflammatory cytokines during the course of the disease, signs of inflammation were found in all three stages of the disease. In an earlier study, during the first two years of CRPS1, about half of the patients showed no inflammatory signs [24], but did fulfil the criteria of Bruehl et al. [15]. Several other studies confirmed that observation, even describing a primarily cold CRPS1 [4, 10, 25, 26].

An explanation for finding a temperature increase and decrease already at the onset of CRPS1 is that blood flow; and thus changes in skin temperature are the results of not only afferent mechanisms, such as local mediators, but also efferent mechanisms. Disturbances in central temperature regulation could result in altered (local) temperature of the injured extremity [27–29]. Recently, we found evidence that temperature changes also might be partly caused by the crosstalk in the vascular system between higher levels of cytokines and the nitric oxide/endothelin-1 (NO/ET-1) balance [30, 31]. Furthermore, blood flow and tissue-blood distribution could be diminished, partly due to disuse of the extremity [13].

A possible explanation for finding edema and atrophy during all stages of the disease is that edema is not only affected by TNF-*α* and IL-6, but also by other inflammatory mediators such as calcitonin gene-related peptide (CGRP) and substance P (SP), and related mechanisms [32, 33].

No correlation was found between the differences in levels of the cytokines and pain and differences between both extremities for temperature, volume, and AROM for all three times of measurement. In an earlier study, we found similar results [24]. Perhaps IL-6 and TNF-*α* are only partially responsible for the permanent damage in CRPS1, expressed as pain, and changes in temperature, volume, and mobility. Other mediators or a combination of mediators, such as nitric oxide [30] and/or amino acids [34, 35] or other mechanisms [7], may also play a role in the pathophysiology of CRPS1 and may explain in part the course of the disease.

## Figures and Tables

**Table 1:** Characteristics of the study population.

Patient characteristic	Value		
Gender: male/female	3/9		
Side: right/left	6/6		
Cause: fracture/accident/surgery/spontaneous	6/2/4/0		
			
	T0	T1	T2
Age in years	52 (48–56)	54 (51–58)	57 (54–62)
Duration of CRPS1 in months	4 (3–14)	35 (21–48)	72 (59–86)
Disease-related medication (number of patients)			
Nonsteroid anti-inflammatory drugs (NSAIDs)	6	1	0
Opiates	4	2	2
Antioxidants	8	1	0
Vasodilators	1	0	0
Muscle relaxants	1	0	0
Antidepressants	1	1	0
Benzodiazepines	2	0	0
Antiepileticum	1	0	1

Data are presented as the *n* or median (interquartile range).

**Table 2:** Levels of IL-6 and TNF-*α* in blister fluid obtained from the CRPS1 and contralateral extremities at first measurement (T0), second measurement (T1), and third measurement (T2). Data are presented as mean (range).

	T0		T1		T2	
	CRPS1	Contralateral	CRPS1	Contralateral	CRPS1	Contralateral
IL-6 (pg/mL)	116* (5–662)	8 (1–36)	80* (1–346)	2 (0–5)	22 (4–78)	20 (3–61)
TNF-*α* (pg/mL)	66* (1–359)	31 (1–258)	56* (3–176)	16 (2–80)	38 (9–81)	47 (10–142)

*Wilcoxon signed ranks test *P* < .05 (CRPS1 versus contralateral).
